# Dumbbell ganglioneuroma of the lumbar spine: a case report and literature review

**DOI:** 10.3389/fsurg.2025.1652850

**Published:** 2025-11-17

**Authors:** Zepeng Xiang, Bao Qi, Chunyang Meng, Qingwei Li

**Affiliations:** 1 Spine Surgery, Affiliated Hospital of Jining Medical University, Jining, Shandong, China; 2 Spine Surgery, Jining Medical University, Jining, Shandong, China; 3 Spine Surgery, China Medical University, Shenyang, China

**Keywords:** ganglioneuroma, lumbar spine, dumbbell-shaped tumor, spinal tumor, case report

## Abstract

**Background:**

Ganglioneuroma (GN) of the lumbar spine is rare, typically occurring in children and young adults. Its diagnosis in middle-aged patients can be challenging. This paper reports a case of a dumbbell-shaped lumbar GN in a middle-aged woman. Case: A 46-year-old woman presented with low back pain and radiating leg pain. MRI revealed a dumbbell-shaped mass at the L2 level. She underwent posterior microscopic total tumor resection with L2–L3 pedicle screw fixation. Postoperative pathology confirmed GN.

**Conclusion:**

At the 1-year follow-up, there was no recurrence. For dumbbell-shaped lumbar GN, a posterior approach combined with internal fixation is an effective strategy. This case suggests that GN should be considered in the differential diagnosis of foraminal masses in middle-aged patients.

## Introduction

Ganglioneuroma (GN) is a rare, well-differentiated, benign tumor originating from neural crest cells, composed of ganglion cells and Schwann cells, and characterized by slow growth (1). It can occur anywhere along the sympathetic chain, is more common in the cervical and thoracic regions, and is relatively rare in the lumbar spine (2). GN is most frequently seen in children and adolescents. Clinical manifestations are non-specific, often involving pain or neurological dysfunction due to nerve root or spinal cord compression, or it may be an incidental finding. Spinal GN accounts for less than 10% of all GNs (3), about 1% of spinal tumors (4), and only 0.8% cause spinal cord compression symptoms (3). Currently, case reports on lumbar GN, particularly dumbbell-shaped lesions in middle-aged patients, remain limited. Such tumors are often misdiagnosed preoperatively as schwannomas, neurofibromas, or lumbar disc herniation. The choice of surgical approach is also challenging due to the tumor's involvement of both intraspinal and extraspinal compartments. Therefore, we report a case of a dumbbell-shaped GN at L2–L3 in a 46-year-old woman. By detailing the clinical presentation, imaging features, surgical management, and outcome, along with a literature review, we aim to enhance clinicians' recognition of this rare entity and provide references for treatment strategy selection.

## Case

A 46-year-old woman presented with a 4-month history of persistent low back pain and radiating pain to the right lower limb. The patient worked as an office clerk and had no significant relevant psychosocial stressors. She denied a family history of neurofibromatosis or other genetic disorders. Her past medical history was unremarkable. The symptoms had gradually worsened, affecting her daily life and work. Preoperatively, she had tried conservative treatment including nonsteroidal anti-inflammatory drugs (NSAIDs) without significant improvement. Physical examination revealed tenderness over the L2–3 paravertebral area, decreased sensation and numbness in the posterolateral region of the right thigh. Muscle strength in the right lower limb was grade 4/5. The femoral nerve stretch test was positive. Knee and ankle jerks were symmetric bilaterally, and pathological signs were negative. Laboratory findings were within normal limits.

**Figure 1:**
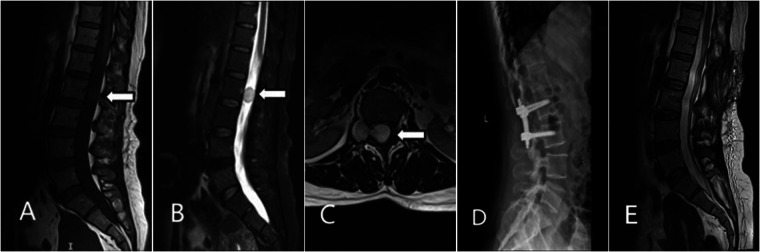
Imaging findings of the dumbbell-shaped tumor at L2–L3. **(A)** Sagittal T1WI showing the intraspinal tumor compressing the thecal sac; **(B)** Sagittal contrast-enhanced T2WI showing heterogeneous enhancement of the tumor; **(C)** Axial T2-weighted image at the L2–L3 level shows the tumor spanning both the intraspinal and extraspinal compartments, demonstrating a dumbbell morphology.; **(D)** Postoperative lateral radiograph demonstrates stable internal fixation at the L2–L3 level.; **(E)** Sagittal T2WI image at 1-year follow-up, showing no signs of recurrence.

Magnetic Resonance Imaging (MRI) showed a dumbbell-shaped abnormal signal mass at the level of the L2 vertebral body, extending laterally through the right neural foramen, which was mildly enlarged. On T2-weighted images (T2WI), the lesion showed heterogeneous high signal intensity with well-defined borders. Contrast-enhanced T1-weighted images revealed mild to moderate heterogeneous enhancement. The lesion measured approximately 33 mm × 17 mm × 17 mm (Figures 1A–C). The remainder of the spinal cord within the canal showed no significant signal abnormalities. Based on clinical and imaging features, the initial radiological diagnosis was a neurogenic tumor, possibly schwannoma or neurofibroma.

Following adequate preoperative preparation, the patient underwent surgery under general anesthesia. A posterior midline approach was utilized for “microscopic resection of an intraspinal tumor with L2–L3 pedicle screw instrumentation”. After dissection of the paraspinal muscles, the L2 and L3 spinous processes and laminae were exposed. The L2 spinous process and bilateral laminae were resected, providing adequate exposure of the tumor and the involved L3 nerve root. Under microscopic visualization, the tumor was noted to be dumbbell-shaped, firm in consistency, and well-encapsulated, with dense adhesion to the L3 nerve root. The tumor was meticulously dissected from the nerve root and the dural boundaries. The entire lesion, including both its intraspinal and extraspinal components, was completely resected. Intraoperatively, a minor dural tear was identified at the site of tumor adhesion. This was meticulously repaired in a tension-free manner using 5-0 polypropylene suture, with no subsequent cerebrospinal fluid leakage observed. Somatosensory evoked potentials (SSEPs) and motor evoked potentials (MEPs) were monitored throughout the procedure and remained stable without significant abnormalities. Given the resection of the L2 spinous process and lamina, pedicle screw instrumentation was placed at the L2 and L3 vertebral bodies to reconstruct spinal stability.

**Figure 2:**
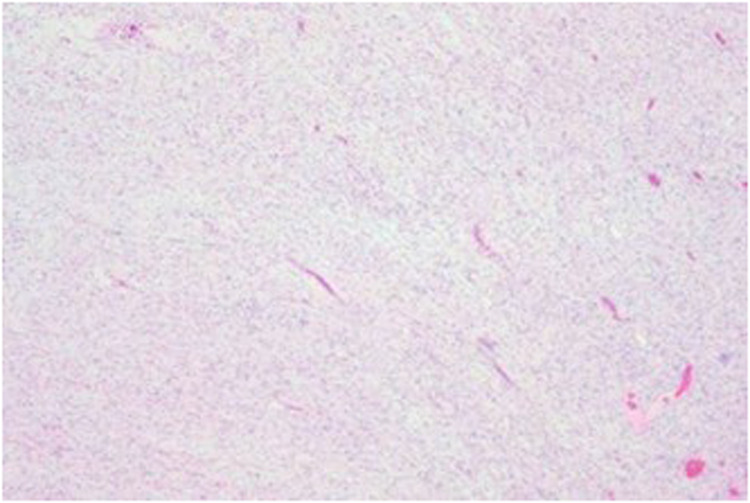
Photomicrograph of the tumor. The tumor consists of ganglion cells with interlacing bundles of spindle cells.

Postoperative pathological examination showed: The tumor tissue was gray-white, gelatinous in texture, and fully encapsulated. Immunohistochemical staining was positive for CD34, S100, and SOX-10. The Ki-67 proliferation index was low (2%–5%) (Figure 2). CD34 positivity suggested vascular richness. Positivity for S100 and SOX-10 supported neural crest origin. The low Ki-67 index (2%–5%) was consistent with benign biological behavior, aligning with GN.

The patient's low back pain and right leg radicular pain significantly improved after surgery. She was able to ambulate with a lumbar brace on postoperative day 2. Postoperative lumbar x-rays showed satisfactory position of the instrumentation and stable spinal alignment (Figure 1D). The sutures were removed one week postoperatively, and she was discharged. At the 1-year outpatient follow-up, the patient was satisfied with the outcome. The severe pre-operative low back and leg pain had essentially resolved, and her quality of daily life was significantly improved (Figure 1E).

## Discussion

Ganglioneuroma (GN) typically occurs in the paraspinal region, but its distribution shows significant segmental variation. For instance, Goldberg et al. (5), in a study of 15 patients with paraspinal GN, noted that these tumors are most common in the thoracic (60%) and sacral (33%) regions, while the lumbar region accounts for only 7%. This distribution is consistent with reports by Sun et al. (2) and Pang et al. (3), indicating that lumbar GN is inherently rare. The present case, located at L2-L3, further confirms the lumbar spine as an uncommon site for GN. Furthermore, GN usually occurs in children and young adults (6, 7). However, the patient in this case was 46 years old, which significantly differs from this typical pattern. Notably, Deora et al.'s review (8) found that cervical GN often presents symptomatically in adulthood due to slow growth, with most cases in their report diagnosed after age 18. Similarly, the median age in Goldberg's series was 30 years (5), and Altalhi et al. (9) reported a case of lumbar GN in a 37-year-old patient. This evidence suggests that although GN is more common in younger individuals, clinicians should still include GN in the differential diagnosis when evaluating foraminal masses in middle-aged or even elderly patients.

Despite typically originating from the paravertebral sympathetic chain and being predominantly located in the epidural space, both the present case and a review of the literature reveal that intradural extension, although rare, is a genuine and clinically significant phenomenon. Early literature reviews by Shephard and Sutton documented such cases, noting their potential to cause severe spinal cord compression (10). It is noteworthy that this intradural invasion is more common in specific populations; a systematic review by Deora et al. found that among reported cases of cervical ganglioneuroma, a significant 62.5% (10/16) exhibited intradural extension (8). Furthermore, intradural extension is a prominent feature in cases associated with Neurofibromatosis Type 1, as illustrated by the case reported by Bacci et al., where tumors demonstrated multifocal, bilateral, symmetric growth throughout the entire spinal axis (11). Therefore, meticulous preoperative evaluation with high-resolution MRI to identify any intradural component is crucial for formulating a surgical strategy aimed at gross total resection and for avoiding intraoperative neural injury.

Clinically, this case presented with low back pain and radiating right leg pain, accompanied by objective neurological deficit (right lower limb muscle strength grade 4/5), consistent with most reports. Symptoms of GN usually result from the mass effect compressing nerve roots or the spinal cord (3, 5, 9). However, a significant proportion of patients are asymptomatic, with the tumor being an incidental finding (5). Additionally, GN can cause scoliosis, particularly with thoracic tumors and in young patients, potentially due to chronic irritation of the vertebral growth plates or paraspinal muscle atrophy (6, 12).

Due to its non-specific clinical presentation, preoperative diagnosis of GN remains challenging. Its imaging features resemble those of more common intraspinal and extraspinal tumors, leading to misdiagnosis (13, 14). The chronic low back pain and radicular symptoms in this case, while typical of lumbar pathology, are non-specific. There are reports in the literature of GN being misdiagnosed as lumbar disc herniation, such as the case described by Shrestha et al. (14), where the preoperative MRI was also interpreted as disc herniation until surgery revealed an L5 nerve root GN. Furthermore, the case reported by Sobowale et al. (13) was radiologically very similar to neurofibroma.

In terms of imaging differentiation, GN typically appears as a mass that is isointense or hypointense on T1WI, hyperintense on T2WI, and shows heterogeneous enhancement after contrast administration. Characteristic features include a dumbbell shape, foraminal enlargement, and smooth pressure erosion of adjacent bone (13, 15, 16). However, these features can also be seen in other neurogenic tumors. Schwannoma is a primary differential diagnosis; it often shows strong homogeneous enhancement, is more prone to cystic degeneration and hemorrhage, and typically involves a single nerve root and foramen (15). Neurofibroma, especially the plexiform type, can also grow in a dumbbell shape, but its borders are often less distinct than those of schwannomas, and it is strongly associated with Neurofibromatosis Type 1 (NF1) (13). Notably, while GN can also be associated with NF1, this is much rarer (13). For large tumors spanning multiple segments, as reported by Wang et al. (16) and in the present case, the possibility of GN should be considered higher, as schwannomas and neurofibromas are usually more localized (9, 16).

Definitive diagnosis relies on histopathological examination. Characteristic findings include the presence of mature ganglion cells within a background of S-100/SOX-10 positive Schwann cells, along with a low Ki-67 proliferation index (13, 16). For atypical or diagnostically challenging cases, CT or ultrasound-guided core needle biopsy can be an effective method to obtain a preoperative pathological diagnosis, aiding in more precise surgical planning (17).

Regarding treatment, gross total resection is the consensus (5, 7). The choice of surgical approach depends on the tumor's anatomical location and extent of involvement. For tumors located predominantly posteriorly or posterolaterally, a posterior or posterolateral approach is often preferred. For example, Rathi et al. (17) described a technique for resecting an L1 nerve root GN through a single posterior incision, involving partial transverse process resection to access the retroperitoneal component without needing an anterior exposure. Conversely, for large tumors located predominantly anterior to the vertebral body, extending into the retroperitoneum, an anterior retroperitoneal approach may be necessary (9). For extensively invasive giant dumbbell tumors, a combined anterior-posterior approach, either staged or simultaneous, may sometimes be required (7).

On the other hand, the role of internal fixation instrumentation in surgery requires careful consideration on a case-by-case basis. When a ganglioneuroma is massive or long-standing, resulting in structural spinal deformities (such as scoliosis) or spinal instability, the use of instrumentation concurrent with tumor resection is both justified and necessary. As reported by Yang et al., this approach effectively corrects deformity and maintains long-term spinal stability (6). However, it is crucial to recognize that for the vast majority of ganglioneuromas not associated with deformity, the primary treatment goal remains complete tumor resection, not the routine use of internal fixation. A large case series by Goldberg et al. confirmed that even patients undergoing subtotal resection—performed to preserve critical neurovascular structures—demonstrate excellent long-term progression-free survival, with favorable outcomes primarily attributed to tumor debulking and decompression (5). Furthermore, Rathi et al. described a modified posterior technique involving resection of the L2 and L3 transverse processes to achieve adequate exposure of the tumor's anterior margin, enabling *en bloc* resection of a large lumbar ganglioneuroma. While instrumentation was utilized in this case, it was primarily to address iatrogenic instability resulting from the requisite bone resection, rather than from the tumor itself (17). Consequently, the surgical strategy must be highly individualized. The application of instrumental fixation should be strictly reserved for cases with pre-existing or anticipated spinal instability and should not be considered a standard component of every surgical procedure for ganglioneuroma.

Based on the anatomical characteristics of the tumor, an individualized treatment plan was developed for this case. The patient is a 46-year-old middle-aged female with a tumor located in the functionally critical lumbar region. To maximize the preservation of postoperative daily living function, a unilateral posterior approach was employed to simultaneously accomplish intraspinal tumor resection and pedicle screw fixation, thereby restoring spinal stability. This treatment strategy aligns with the experiences reported by Wang et al. (16) and Goldberg et al. (5). Furthermore, given the benign nature of GN, chemotherapy and radiotherapy have limited roles following tumor resection (10).

Regarding postoperative prognosis, the literature consistently indicates an excellent long-term outcome for GN. Goldberg's study showed no evidence of tumor recurrence or progression in any patient after a median follow-up of 68 months, with residual tumors after subtotal resection also remaining stable (5, 18). The present case showed no recurrence and good functional recovery at the 1-year follow-up, consistent with the literature consensus (3, 5, 9). Additionally, the successful intraoperative management of dural adhesion and the use of intraoperative neurophysiological monitoring in this case highlight the importance of preserving neurological function in modern spinal tumor surgery (8, 16).

## Conclusion

Dumbbell-shaped ganglioneuroma of the lumbar spine is clinically rare and diagnostically challenging preoperatively. This case confirms that for dumbbell-shaped tumors involving load-bearing lumbar segments, posterior microscopic gross total resection combined with single-stage internal fixation can achieve good oncological control and spinal stability. By presenting GN in the atypical context of a middle-aged patient with a lumbar lesion, this case expands the clinical spectrum of this entity. It also demonstrates the feasibility of a single posterior approach for managing such complex tumors involving critical spinal segments, providing a reference for clinical diagnosis and treatment.

## Data Availability

The original contributions presented in the study are included in the article/Supplementary Material, further inquiries can be directed to the corresponding author.
